# LncRNA MAGI2-AS3 inhibits bladder cancer progression by targeting the miR-31-5p/TNS1 axis

**DOI:** 10.18632/aging.104162

**Published:** 2020-11-20

**Authors:** Congyi Tang, Yi Cai, Huichuan Jiang, Zhengtong Lv, Changzhao Yang, Haozhe Xu, Zhi Li, Yuan Li

**Affiliations:** 1 Department of Urology, Xiangya Hospital, Central South University, Changsha 410008, Hunan, China

**Keywords:** bladder cancer, MAGI2-AS3, miR-31-5p, TNS1

## Abstract

In this study, we performed bioinformatics analysis to identify the competing endogenous RNAs (ceRNAs) that regulate bladder cancer (BCa) progression. RNA-sequencing data analysis identified 2451 differentially expressed mRNAs, 174 differentially expressed lncRNAs, and 186 microRNAs (miRNAs) in BCa tissues (n=414) compared to the normal urothelial tissues (n=19) from the TGCA database. CeRNA network analysis of the differentially expressed lncRNAs and mRNAs showed strong positive correlation between lncRNA MAGI2-AS3 and Tensin 1 (TNS1) mRNA in BCa tissues. Bioinformatics analysis also showed that both MAGI2-AS3 and TNS1 mRNA sequences contain miR-31-5p binding sites. Furthermore, we observed significantly lower MAGI2-AS3 and TNS1 mRNA expression and higher miR-31-5p expression in the BCa tissues and cell lines (T24 and J82) compared with their corresponding controls. Functional and biochemical experiments in BCa cell lines including luciferase reporter assays showed that MAGI2-AS3 upregulated TNS1 by sponging miR-31-5p. Transwell assays showed that the MAGI2-AS3/miR-31-5p/TNS1 axis regulated migration and invasion ability of BCa cell lines. Moreover, immunohistochemical staining of paired BCa and normal urothelial tissues showed that low expression of TNS1 correlated with advanced tumor (T) stages and lymph node metastasis in BCa. In conclusion, our study demonstrates that the MAGI2-AS3/miR-31-5p/TNS1 axis regulates BCa progression.

## INTRODUCTION

Bladder cancer (BCa) is the 10^th^ most common cancer with 549,000 new cases and 200,000 deaths reported worldwide in 2018 [1]. In the United States, 81400 new cases and 17980 BCa-related deaths are projected for 2020 [2]. The standard treatments for BCa patients include surgery, radiotherapy and chemotherapy, but, the prognosis of patients with advanced-stage BCa remains poor [3]. Therefore, new diagnostic and prognostic biomarkers are required to guide early diagnosis and effective treatments of BCa patients to improve their survival outcomes.

Several reports have shown that competing endogenous RNAs (ceRNAs) such as small non-coding RNAs, pseudogenes, long noncoding RNAs (lncRNAs) and circular RNAs (circRNAs) modulate expression of their target genes by binding to microRNAs (miRNAs) [4–7]. LncRNAs are transcripts without any protein-coding potential that are approximately 200 nucleotides in length and act by sponging miRNAs and enhancing the expression of their target mRNAs [8–10]. The regulatory role of lncRNAs has been widely studied in BCa. For example, lncRNA DANCR enhances the malignancy of BCa cells by sponging miR-149 and increasing the expression of MSI2 [11]. LncRNA SPRY4-IT1 promotes proliferation and metastasis of BCa cells by sponging miR-101-3p and increasing the expression of EZH2 [12].

Tensin 1 (TNS1) is a focal adhesion molecule that has been implicated in the migration of normal and tumor cells [13]. The role of TNS1 is controversial in different cancers. Low expression of TNS1 promotes metastasis and invasion of prostate cancer and breast cancer cells [14, 15]. However, high expression of TNS1 promotes metastasis and invasion of colorectal cancer cells [16, 17]. In this study, we performed bioinformatics analyses and functional *in vitro* experiments to determine the regulatory mechanisms underlying the expression of TNS1 in BCa tissues and cell lines.

## RESULTS

### LncRNA MAGI2-AS3 and TNS1 mRNA expression is decreased and miR-31-5p expression is upregulated in BCa tissues from the TGCA database

**Figure 1:**
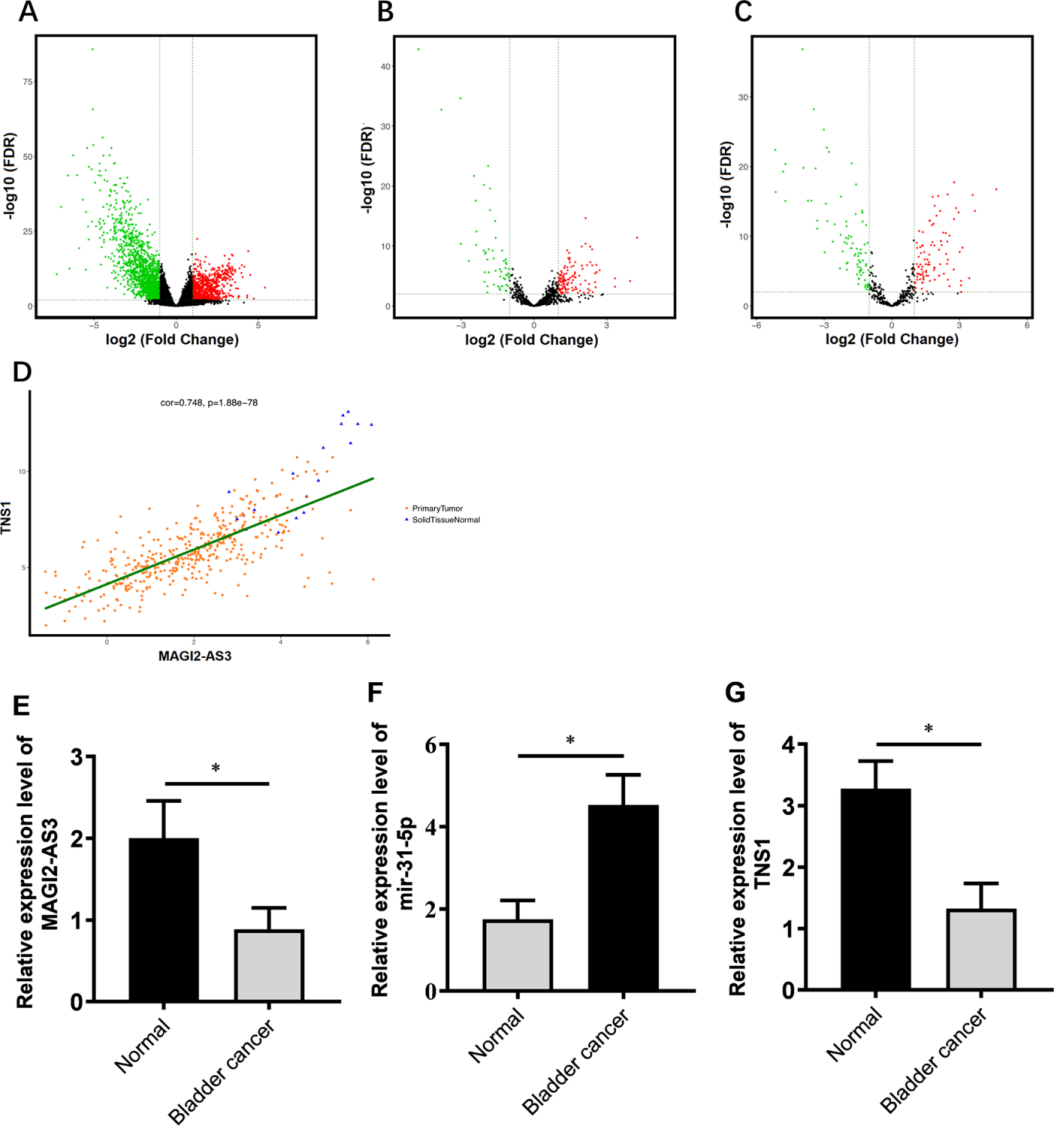
**Correlation analysis of MAGI2-AS3, miR-31-5p and TNS1 mRNA expression in TGCA-BCa patient tissues.** (**A**) Volcanic map of differentially expressed mRNAs in BCa tissues compared to normal urothelial tissues. (**B**) Volcanic map of differentially expressed lncRNAs in BCa tissues compared to normal urothelial tissues. (**C**) Volcanic map of differentially expressed miRNAs in BCa tissues compared to normal urothelial tissues. Note: p<0.05 and logFC>2 were used as threshold parameters. Green dots represent down-regulated RNAs, and red dots represent up-regulated RNAs. (**D**) Distribution map shows correlation between MAGI2-AS3 and TNS1 expression in BCa patients from the TGCA database. Orange dots represent primary BCa samples and blue triangles represent normal urothelial tissue samples. (**E**–**G**) Histogram plots show the expression of MAGI2-AS3, mir-31-5p and TNS1 in BCa and normal bladder tissues in the TCGA database. The analyses were performed using the R software. * denotes p<0.05 and **denotes p<0.01.

We analyzed the RNA-sequencing data from 412 patients with BCa and normal bladder tissues using log_2_FC >2.0 and adjusted P value (FDR) < 0.01 as threshold parameters to identify differentially expressed mRNAs, lncRNAs, and miRNAs. The volcano maps of differentially expressed lncRNAs, miRNAs, and mRNAs are shown in Figure 1A–1C. We identified 2451 differentially expressed mRNAs (818 up-regulated and 1633 down-regulated; Supplementary Table 3), 174 differentially expressed lncRNAs (118 up-regulated and 56 down-regulated; Supplementary Table 1) and 186 differentially expressed miRNAs (88 up-regulated and 98 down-regulated; Supplementary Table 2) in the BCa tissues compared to the normal bladder tissues.

Next, we constructed a ceRNA regulatory network to identify functionally significant interactions between differentially expressed lncRNAs, miRNA and mRNAs in BCa tissues. We analyzed the correlations between differentially expressed lncRNAs and mRNAs using GDCRNATools and shinyCorPlot functions in R language and ranked lncRNA-mRNA pairs as shown in Table 1. The correlation between MAGI2-AS3 and TNS1 was ranked second. Since the functional relationship between MAGI2-AS3 and TNS1 has not been investigated in BCa, we chose them for further investigation. The distribution map of MAGI2-AS3 and TNS1 in BCa patients is shown in Figure 1D.

**Table 1:** Expression correlation between mRNA and lncRNA.

**lncRNA**	**mRNA**	**cor**	**P value**
MAGI2-AS3	ZEB2	0.786	2.85E-85
MAGI2-AS3	*TNS1*	0.749	8.76E-79
MAGI2-AS3	MYLK	0.743	5.51E-77
MAGI2-AS3	KLF9	0.726	6.54E-73
MAGI2-AS3	RECK	0.718	1.07E-69
MAGI2-AS3	ZCCHC24	0.711	7.24E-68
MAGI2-AS3	ATP8B2	0.69	2.57E-62
MAGI2-AS3	LRCH2	0.69	1.76E-62
MAGI2-AS3	TCF4	0.684	5.47E-61
MAGI2-AS3	STARD13	0.677	2.36E-59
MAGI2-AS3	PDE4B	0.652	1.23E-53
MAGI2-AS3	CYBRD1	0.647	1.06E-52
MAGI2-AS3	AKT3	0.639	5.44E-51
MAGI2-AS3	EPB41L2	0.626	1.64E-48
MAGI2-AS3	PRR16	0.62	2.78E-47
MAGI2-AS3	RUSC2	0.593	1.74E-42
MAGI2-AS3	DMD	0.587	1.87E-41
MAGI2-AS3	EBF3	0.582	1.27E-40
MAGI2-AS3	EDIL3	0.577	7.29E-40
MAGI2-AS3	LATS2	0.552	6.58E-36
AC093010.3	CNTNAP1	0.544	9.96E-35
AC093010.3	ZCCHC24	0.534	2.02E-33
AC074117.1	KIFC2	0.528	1.68E-32
AC074117.1	MEX3A	0.511	3.18E-30
AC093010.3	LPP	0.508	6.69E-30

cor = correlation coefficient.

We identified miR-31-5p as the top ranked putative target of MAGI2-AS3 with a complementary strand sequence, 5’ AUCUUGCC-UAGAACGG-3’ (Supplementary Table 4). Starbase analysis showed that TNS1 was a potential downstream target gene of miR-31-5p (Supplementary Table 5). The analysis of BCa patient tissues from the TGCA database showed that the expression of MAGI2-AS3 and TNS1 was significantly downregulated and miR-31-5p was significantly upregulated in BCa tissues compared to the normal urothelial tissues (Figure 1E–1G).

### MAGI2-AS3, miR-31-5p and TNS1 expression levels in BCa tissues and cell lines

**Figure 2:**
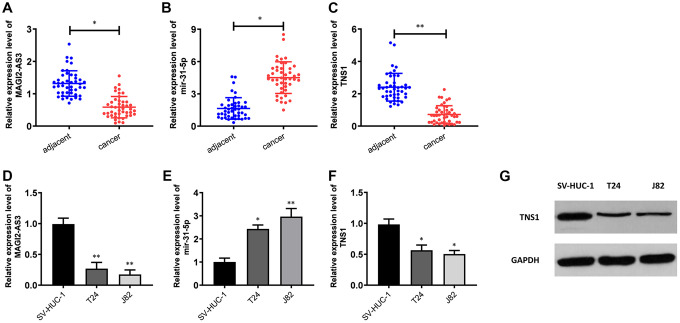
**Expression levels of MAGI2-AS3, miR-31-5p and TNS1 in BCa tissues and cell lines.** (**A**–**C**) QRT-PCR analysis results show the expression levels of MAGI2-AS3, miR-31-5p and TNS1 in BCa and adjacent normal tissues (n=45). (**D**–**F**) QRT-PCR analysis results show the expression levels of MAGI2-AS3, mir-31-5p and TNS1 in the normal urothelial cell line (SV-HUC-1) and BCa cell lines (T24 and J82). (**G**) Western blot analysis shows the expression of TNS1 in the normal urothelial cell line (SV-HUC-1) and BCa cell lines (T24 and J82). GAPDH was used as an internal control. *p<0.05. **p<0.01.

We performed qRT-PCR analysis to verify the expression of MAGI2-AS3, miR-31-5p and TNS1 in 45 pairs of BCa and adjacent normal urothelial tissues. QRT-PCR results showed that MAGI2-AS3 and TNS1 levels were down-regulated and miR-31-5p levels were up-regulated in BCa tissues compared to the adjacent normal urothelial tissues (Figure 2A–2C). Moreover, MAGI2-AS3 and TNS1 levels were significantly reduced and miR-31-5p levels were increased in the BCa cell lines (T24 and J82) compared to the normal urothelial cell line, SV-HUC-1 (Figure 2D–2F). Furthermore, western blot analysis showed that TNS1 protein expression was significantly downregulated in the BCa cell lines compared to the normal urothelial cell line, SV-HUC-1 (Figure 2G). These results demonstrate the regulatory relationship between MAGI2-AS3, miR-31-5p and TNS1 in BCa tissues.

### MAGI2-AS3 promotes TNS1 expression by sponging miR-31-5p

**Figure 3:**
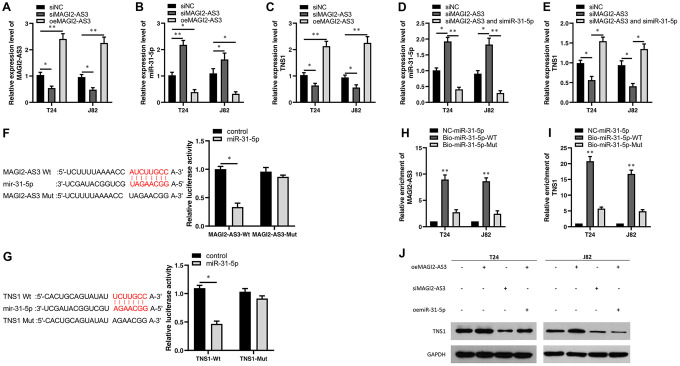
**Characterization of the regulatory relationship between MAGI2-AS3, mir-31-5p and TNS1 in BCa cells.** (**A**–**C**) QRT-PCR analysis shows the expression levels of MAGI2-AS3, miR-31-5p and TNS1 mRNA in control (si-NC-transfected), MAGI2-AS3 knockdown (siMAGI2-AS3-transfected) and MAGI2-AS3-overexpressing (oeMAGI2-AS3) T24 and J82 cell lines. (**D**, **E**) QRT-PCR analysis shows miR-31-5p and TNS1 mRNA levels in control, MAGI2-AS3-silenced, and MAGI2-AS3-silenced plus miR-31-5p-silenced T24 and J82 cell lines. (**F**) Luciferase reporter assay results show the relative luciferase activity in T24 cells transfected with vectors containing wild-type MAGI2-AS3 and control or miR-31-5p compared to those transfected with vectors containing mutant MAGI2-AS3 and control or miR-31-5p. (**G**) Luciferase reporter assay results show the relative luciferase activity in T24 cells transfected with vectors containing wild-type TNS1 and control or miR-31-5p compared to those transfected with mutated TNS1 and control or miR-31-5p. (**H**, **I**) RNA pull-down assay results show the relative levels of MAGI2-AS3 and TNS1 pulled down by NC-miR-31-5p, Bio-miR-31-5p-WT or Bio-miR-31-5p-Mut. (**J**) Representative western blot shows TNS1 protein levels in control, MAGI2-AS3 knockdown (siMAGI2-AS3-transfected), MAGI2-AS3-overexpressing (oeMAGI2-AS3), and MAGI2-AS3-overexpressing plus miR-31-5p-overexpressing (oeMAGI2-AS3 plus oemiR-31-5p) T24 and J82 cells. *p<0.05. **p<0.01. GAPDH was used as internal control.

Next, we transfected siRNA against MAGI2-AS3 (si-MAGI2-AS3) in T24 and J82 cells and analyzed the expression levels of miR-31-5p and TNS1 compared to corresponding controls. MAGI2-AS3 levels were significantly reduced in the siMAGI2-AS3-transfected T24 and J82 cells compared to the siNC-transfected T24 and J82 cells (Figure 3A). Moreover, miR-31-5p levels were upregulated and TNS1 levels were downregulated in the siMAGI2-AS3-transfected BCa cells compared to the si-NC-transfected BCa cells (Figure 3B, 3C). We then overexpressed MAGI2-AS3 in the T24 and J82 cells and observed significant reduction in miR-31-5p levels and significant upregulation of TNS1 (Figure 3B, 3C). However, si-miR-31-5p plus si-MAGI2-AS3-transfected T24 and J82 cells showed significant downregulation of miR-31-5p (Figure 3D) and upregulation of TNS1 (Figure 3E).

We then performed luciferase reporter assays to verify direct interactions between MAGI2-AS3 and miR-31-5p as well as miR-31-5p and TNS1 mRNA. The results showed that miR-31-5p mimics decreased the luciferase activity of T24 cells transfected with vectors containing wild-type MAGI2-AS3 and wild-type TNS1, but did not affect the luciferase activity in T24 cells transfected with mutated MAGI2-AS3 and mutated TNS1-mut constructs (Figure 3F, 3G). RNA pull-down experiments showed that biotinylated wild-type miR-31-5p (Bio-miR-31-5p-WT) pulled down significant amounts of MAGI2-AS3 and TNS1 from BCa cells compared to non-biotinylated miR-31-5p (NC-miR-31-5p) and biotinylated mutant miR-31-5p (Bio-miR-31-5p-Mut) (Figure 3H, 3I).

Moreover, western blot analysis showed that TNS1 protein levels were significantly upregulated in the MAGI2-AS3-OE group and downregulated in the siMAGI2-AS3 group (Figure 3J). However, miR-31-5p overexpression reduced the levels of TNS1 protein in the MAGI2-AS3 overexpressing T24 and J82 cells (Figure 3J). These results demonstrate a direct regulatory relationship between MAGI2-AS3, miR-31-5p and TNS1.

### MAGI2-AS3 inhibits proliferation, migration and invasion of BCa cells

**Figure 4:**
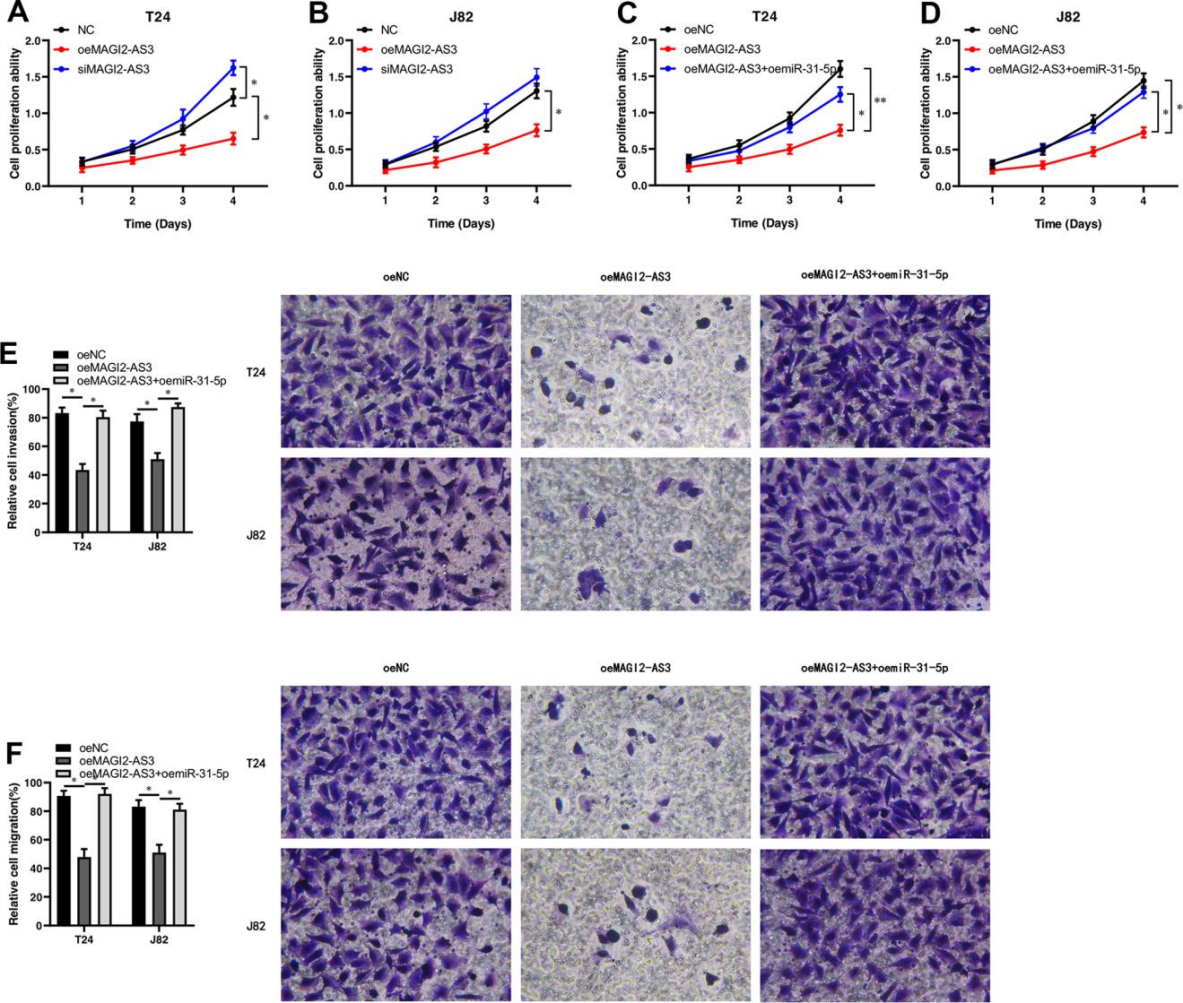
**MAGI2-AS3 and miR-31-5p regulate proliferation, invasion and migration of BCa cells.** (**A**, **B**) MTT assay results show the proliferation status of control (si-NC-transfected), MAGI2-AS3 knockdown (siMAGI2-AS3-transfected) and MAGI2-AS3-overexpressing (oeMAGI2-AS3) T24 and J82 cells. (**C**, **D**) MTT assay results show the proliferation status of control (oeNC), MAGI2-AS3-overexpressing (oeMAGI2-AS3), and MAGI2-AS3-overexpressing plus miR-31-5p-overexpressing (oeMAGI2-AS3 plus oemiR-31-5p) T24 and J82 cells. (**E**, **F**) Transwell assay results show the invasiveness and migration ability of control (oeNC), MAGI2-AS3-overexpressing (oeMAGI2-AS3), and MAGI2-AS3-overexpressing plus miR-31-5p-overexpressing (oeMAGI2-AS3 plus oemiR-31-5p) T24 and J82 cells. *p<0.05, **p<0.01.

We analyzed the role of the MAGI2-AS3/miR-31-5p/TNS1 axis on the proliferation of BCa. MTT assays showed that MAGI2-AS3 overexpression inhibited BCa cell proliferation, whereas, MAGI2-AS3 silencing enhanced BCa cell proliferation (Figure 4A, 4B). Moreover, miR-31-5p overexpression increased the proliferation of MAGI2-AS3 overexpressing BCa cells compared to the corresponding controls (Figure 4C, 4D). Transwell migration and invasion assays showed that MAGI2-AS3 over-expression significantly reduced the numbers of migrating and invading BCa cells, whereas, miR-31-5p overexpression increased the migration and invasiveness of MAGI2-AS3 overexpressing BCa cells (Figure 4E, 4F). After siMAGI2-AS3, there was no significant difference in migration and invasion, possibly because the expression of MAGI2-AS3 in T24 and J82 cells was already very low. Overall, our results demonstrate that the MAGI2-AS3/miR-31-5p/TNS1 axis regulates proliferation, migration, and invasiveness of BCa cells.

### TNS1 expression is down-regulated in BCa tissues and negatively correlates with cancer progression

**Figure 5:**
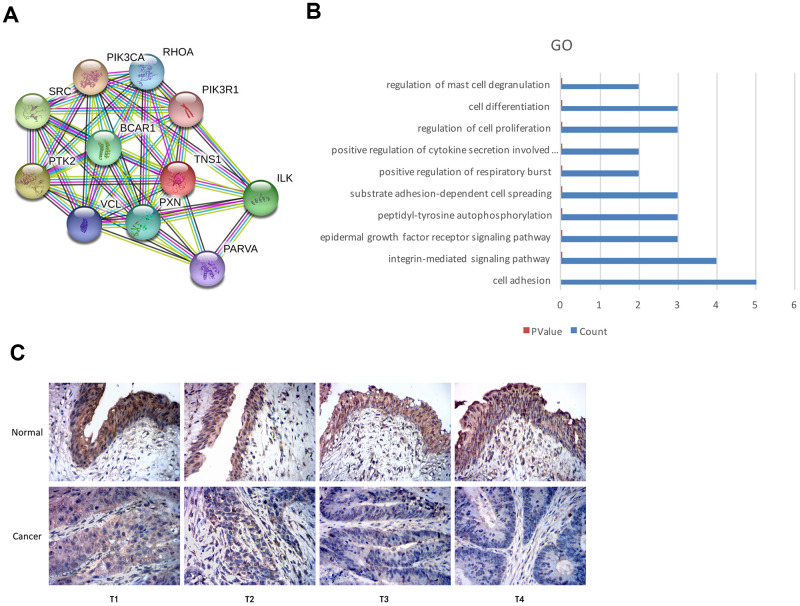
**Top GO terms associated with TNS1 and immunohistochemical analysis of TNS1 expression in BCa and normal urothelial tissues.** (**A**) STRING database analysis shows the protein interaction network between TNS1 and TNS1-related proteins. The nodes represent proteins and the lines represent protein-protein interactions. (**B**) Top 10 GO terms related to TNS1 and TNS1-related proteins based on functional enrichment analysis using DAVID. The blue bars represent the number of TNS1-related genes in each GO term. (**C**) Immunohistochemical analysis results show TNS1 protein expression in BCa and adjacent normal urothelial tissues from T1, T2, T3 and T4 stage BCa patients.

We used the STRING database and identified proteins interacting with TNS1 (Figure 5A). Functional enrichment analysis of TNS1 and TNS1-related proteins showed enrichment of cell adhesion pathway (Figure 5B). Tensin is a family of proteins involved in focal adhesion and includes four members, namely, tensin1 (TNS1), tensin2 (TNS2), tensin3 (TNS3) and C-terminal tensin-like (CTEN) [18–20]. TNS1 is a focal adhesion molecule that binds the actin cytoskeleton to the integrins and also forms a signaling complex through its multiple binding domains [19, 20]. We postulated that TNS1 may be related to tumor progression in BCa. Therefore, we analyzed the expression of TNS1 in 45 pairs of BCa and adjacent normal urothelial tissues at different T stages and the results are summarized in Table 2. Overall, the expression of TNS1 was significantly lower in the BCa tissues compared to normal bladder tissues; moreover, the expression of TNS1 was significantly lower in the advanced T stages (T3-4) compared to the patients in the lower T stages (T1-2) (Figure 5C). Based on the chi-square test, we found low expression of TNS1 correlated with lymph node metastasis in BCa patients (Table 2). Therefore, our results demonstrate that low TNS1 expression correlates with tumor progression and poor prognosis in BCa patients.

**Table 2:** Correlation between *TNS1* expression and clinicopathological factors.

**Characteristics**		**Total**	***TNS1* Expression**		**p-value**
			**Low**	**High**	
Specimen source	Cancer tissue	45	33	12	1.21E-06
	Normal tissue	45	10	35	
Gender	Male	32	23	9	0.7285
	Female	13	10	3	
Age	<60	21	14	7	0.3441
	≥60	24	19	5	
Primary tumor	T1-T2	18	10	8	0.0277
	T3-T4	27	23	4	
Nodal metastasis	N0	25	15	10	0.0237
	N1-N2	20	18	2	
Distant metastasis	M0	32	22	10	0.2753
	M1	13	11	2	

χ2 test, p < 0.05 is considered to be of statistically significant.

## DISCUSSION

LncRNA are non-protein coding RNA molecules that are ≥200 nucleotides in length and play significant roles in critical cellular functions under normal physiological and pathophysiological conditions [21, 22]. Several lncRNAs have been identified as diagnostic and prognostic biomarkers in many cancer types. For example, lncRNA PCA3 is highly expressed in prostate cancer [23]. PCA3 levels in urine show higher sensitivity in prostate cancer diagnosis than the standard PSA tests [24, 25]. LncRNA MALAT1 expression inversely correlates with the progression and metastasis of breast cancer [26, 27].

The roles of several lncRNAs have been investigated in BCa [28–33]. UCA1 is very sensitive and specific for rhe diagnosis of BCa [29], and it can also affect the progress of BCa [30, 31]. TUG1 can mediate cell proliferation affect the metastasis and grading of BCa [32, 33]. In this study, we demonstrate that lncRNA MAGI2-AS3 expression was significantly reduced in BCa tissues compared to the normal bladder tissues. Moreover, we demonstrate that the MAGI2-AS3/miR-31-5p/TNS1 axis regulates proliferation, metastasis and invasion of BCa cells. The role of MAGI2-AS3 has also been investigated in other cancers. MAGI2-AS3 inhibits invasion and metastasis of breast cancer cells by sponging mir-374a [34]. MAGI2-AS3 also regulates proliferation and metastasis of hepatocellular carcinoma cells through the mir-374b-5p/SMG1 axis [35].

Tensin protein family members regulate metastasis in many cancers [18]. TNS1 is involved in cell migration and invasion [36]. TNS1/miR-548j axis regulates invasion and metastasis of breast cancer cells [14]. TNS1 expression also correlates with bone metastasis of prostate cancer [15]. In the present study, we demonstrate that low expression of TNS1 is associated with advanced T stages and lymph node metastasis. Therefore, TNS1 is a potential prognostic biomarker for BCa.

In conclusion, our study demonstrates that the MAGI2-AS3/miR-31-5p/TNS1 axis regulates BCa progression. Hence, MAGI2-AS3, miR-31-5p, and TNS1 are potential prognostic biomarkers that can predict survival outcomes of BCa patients. Further studies are needed to verify the findings of our study.

## MATERIALS AND METHODS

### Data retrieval from the TCGA database

We searched the TCGA database (https://portal.gdc.cancer.gov) using TCGA-BLCA as project ID and downloaded clinical data for 412 BCa patients. We also downloaded RNA-seq data for 414 primary BCa and 19 normal urothelial tissue samples, and miRNA-seq data for 417 primary BCa and 19 normal urothelial tissue samples.

### Identification of differentially expressed mRNAs and lncRNAs

We removed the duplicate samples, non-primary tumor and non-solid normal tissue samples from the RNA-seq and miRNA-seq datasets. Then, we integrated and normalized the gene expression data of the BCa patients in comparison to normal urothelial tissues by using the calcNormFactors function and screened for differentially expressed mRNAs and lncRNAs using the R programming language.

### Construction of the regulatory network between ceRNAs

The potential interactions between lncRNAs and miRNAs were identified using miRcode (http://www.mircode.org) and Starbase (http://starbase.sysu.edu.cn) databases. The interactions between miRNAs and mRNAs were predicted with the miRTarBase (http://mirtar.mbc.nctu.edu.tw/human/), miRDB (http://mirdb.org) and TargetScan (http://www.targetscan.org/vert_72/) databases.

### Target gene prediction and functional enrichment analysis

We used the Starbase database to determine the direct relationship between lncRNAs and miRNAs as well as miRNAs and mRNAs, and the target binding sites in each pair. We used the STRING (https://string-db.org) database to identify related proteins and performed functional enrichment analysis using DAVID (https://david.ncifcrf.gov).

### Clinical samples from BCa patients

We obtained 45 pairs of cancer and adjacent normal tissues from 45 BCa patients who underwent radical cystectomy at the Xiangya Hospital. These patients had not received any radiotherapy or chemotherapy before surgery. The tissues were stored in liquid nitrogen before analysis. This study was approved by the Ethics committee of Xiangya Hospital. We obtained signed written informed consent from all patients.

### Cell culture

The human normal urothelial cell line (SV-HUC-1) and BCa cell lines (T24 and J82) were purchased from the Cell Bank of the Chinese Academy of Sciences (Shanghai, China) and cultured with Dulbecco modified Eagle medium (DMEM) containing 10% fetal bovine serum (FBS) in a humidified chamber at 37° C and 5% CO2.

### Quantitative real time PCR

We extracted total cellular or tissue RNA using TRlzol reagent (Thermo Fisher Scientific, Shanghai, China). Equal amounts of total RNAs were reverse transcribed into cDNA using the PCR kit (Takara, Dalian, China). Then, q-PCR was performed in the ABI7500 Real-Time PCR system and the expression of MAGI2-AS3, TNS1 and miR-31-5p was evaluated using the 2^-ΔΔCt^ method relative to corresponding controls.

### Generation of MAGI2-AS3 silenced BCa cells

We knocked down the expression of MAGI2-AS3 by transfecting BCa cell lines with siRNAs against MAGI2-AS3 (Genepharma, Suzhou, China). Briefly, 2 × 10^5^ BCa cells were seeded in each well in 6-well plates and transfected with si-NC (control) or si-MAGI2-AS3 using lipofectamine for 48 h according to manufacturer’s instructions. The efficiency of MAGI2-AS3 knockdown was analyzed by qRT-PCR.

### Generation of MAGI2-AS3 overexpressing BCa cells

We first cloned the MAGI2-AS3 DNA sequence into the GV358 lentiviral vector (Genechem, Shanghai, China). We then co-transfected the 293T cells with oeMAGI2-AS3 vector plasmid and helper plasmids for 48 h. We collected the supernatants and purified the lentiviruses carrying the oeMAGI2-AS3 plasmid by centrifugation and further concentrated the virus through ultrafiltration. The ultrapure oeMAGI2-AS3 plasmid and polybrene was incubated with T24 and J82 cells (2 × 10^5^ cell/ml) for 48 h. The efficiency of MAGI2-AS3 overexpression in the BCa cells was confirmed by observing the GFP fluorescence under a fluorescence microscope.

### RNA pull-down assay

RNA pull-down assay was performed using the Pierce Magnetic RNA-Protein Pull-Down Kit (Thermo Fisher Scientific, Waltham, MA, USA). The biotinylated MAGI2-AS3 (Bio-MAGI2-AS3), biotinylated miRNAs (Bio-miR-31-5p-WT and Bio-miR-31-5p-Mut) and the corresponding controls (NC-MAGI2-AS3 and NC-miR-31-5p) were synthesized by GenePharma (Suzhou, China). Briefly, BCa cell lysates were prepared using the RIPA lysis buffer and incubated with the control and biotinylated RNAs. The relative levels of the RNAs (MAGI2-AS3, miR-31-5p, or TNS1 mRNA) were detected using qRT-PCR.

### Western blotting

We prepared total protein lysates using the RIPA lysis buffer and quantified the proteins using the BCA protein assay. Equal amounts of total cell protein lysates were separated by 10% sodium dodecyl sulfate (SDS) polyacrylamide gel electrophoresis and transferred onto the polyvinylidene fluoride (PVDF) membranes. The membranes were blocked with 5% skimmed milk for 10 minutes. Then, the membranes were incubated overnight with the primary antibody against TNS1 (Proteintech, Wuhan, China) at 4 °C. Then, the membranes were incubated with secondary antibodies (Proteintech, Wuhan, China). The blots were developed using ECL and the protein bands were captured on a film through scanning. The integrated optical density (IOD) value of the protein bands were evaluated using Total Lab Quant V11.5 (Newcastle upon Tyne, UK,)

### Luciferase reporter assay

We PCR amplified the miR-31-5p sequence containing binding sites for wild-type MAGI2-AS3 and TNS1 according to the Starbase and cloned into the pGL3 reporter plasmid (Promega, Beijing, China). Then we constructed target plasmids containing wild-type or mutant MAGI2-AS3 and TNS1 constructs (Thermo Fisher Scientific, Shanghai, China). We cultured T24 cells in a 6-well plate for 24 h, and then transfected them with different combinations of reporter and target plasmids for 48 h. We used the GloMax 20/20 fluorescence detector (Promega, Beijing, China) to detect the fluorescence intensity of the reporter gene plasmid.

### MTT cell proliferation assay

We incubated 5 × 10^3^ cells / well in 96-well plates for 24, 48, 72, and 96 h. When the incubation time was reached, we added 10 μl of MTT solution into each well and incubated further for 4 h. We then added 150 μl DMSO to dissolve the crystals in each well, and measured the absorbance at 490 nm in a Fluoroskan microplate reader (Thermo Fisher Scientific, Shanghai, China).

### Transwell migration and invasion assay

To determine the BCa cell migration ability, we added 5 × 10^4^ T24 and J82 cells in 500 μl DMEM without FBS into the upper chambers and 500 μl DMEM medium with 20% FBS into the lower chamber and incubated the Transwell chambers in a humidified incubator at 5% CO_2_ and 37° C for 48 h. Then, the cells in the upper chamber were removed and the cells in the lower chamber were fixed with methanol for 15-20 minutes, and stained with 0.1% crystal violet for 15 minutes. The average numbers of cells in five randomly selected areas were counted for each sample under a light microscope to determine their cell migration ability. To determine the invasive ability of the cells, we added Matrigel (ECM) between the Transwell chambers and repeated the experiment as described for the migration assay.

### Immunohistochemistry (IHC)

The sections of BCa and normal adjacent tissues were deparaffinized and permeabilized. Then, the specimens were incubated with the anti-TNS1 antibody (Proteintech, Wuhan, China) overnight at 4^o^C. Then, we incubated the specimens with the secondary antibody (Proteintech, Wuhan, China). The color was developed with DBA and the stained samples were imaged with a Nikon E200 microscope.

### Statistical analysis

All experiments were performed at least in triplicates. The data are expressed as means ± standard deviation (S.D). Differences between samples were evaluated using two-tailed t-tests. Values of P < 0.05 were considered statistically significant. GraphPad software is used to determine the P value.

## Supplementary Material

Supplementary Tables

## References

[1] Bray F, Ferlay J, Soerjomataram I, Siegel RL, Torre LA, Jemal A. Global cancer statistics 2018: GLOBOCAN estimates of incidence and mortality worldwide for 36 cancers in 185 countries. CA Cancer J Clin. 2018; 68:394–424. 10.3322/caac.21492 30207593

[2] Siegel RL, Miller KD, Jemal A. Cancer statistics, 2020. CA Cancer J Clin. 2020; 70:7–30. 10.3322/caac.21590 31912902

[3] Miyamoto DT, Mouw KW, Feng FY, Shipley WU, Efstathiou JA. Molecular biomarkers in bladder preservation therapy for muscle-invasive bladder cancer. Lancet Oncol. 2018; 19:e683–95. 10.1016/S1470-2045(18)30693-4 30507435

[4] Thomson DW, Dinger ME. Endogenous microRNA sponges: evidence and controversy. Nat Rev Genet. 2016; 17:272–83. 10.1038/nrg.2016.20 27040487

[5] Tay Y, Rinn J, Pandolfi PP. The multilayered complexity of ceRNA crosstalk and competition. Nature. 2014; 505:344–52. 10.1038/nature12986 24429633 PMC4113481

[6] Wang J, Liu X, Wu H, Ni P, Gu Z, Qiao Y, Chen N, Sun F, Fan Q. CREB up-regulates long non-coding RNA, HULC expression through interaction with microRNA-372 in liver cancer. Nucleic Acids Res. 2010; 38:5366–83. 10.1093/nar/gkq285 20423907 PMC2938198

[7] Robertson AG, Kim J, Al-Ahmadie H, Bellmunt J, Guo G, Cherniack AD, Hinoue T, Laird PW, Hoadley KA, Akbani R, Castro MA, Gibb EA, Kanchi RS, et al, and TCGA Research Network. Comprehensive molecular characterization of muscle-invasive bladder cancer. Cell. 2018; 174:1033. 10.1016/j.cell.2018.07.036 30096301 PMC6297116

[8] Iyer MK, Niknafs YS, Malik R, Singhal U, Sahu A, Hosono Y, Barrette TR, Prensner JR, Evans JR, Zhao S, Poliakov A, Cao X, Dhanasekaran SM, et al. The landscape of long noncoding RNAs in the human transcriptome. Nat Genet. 2015; 47:199–208. 10.1038/ng.3192 25599403 PMC4417758

[9] Schmitt AM, Chang HY. Long noncoding RNAs in cancer pathways. Cancer Cell. 2016; 29:452–63. 10.1016/j.ccell.2016.03.010 27070700 PMC4831138

[10] Wang KC, Chang HY. Molecular mechanisms of long noncoding RNAs. Mol Cell. 2011; 43:904–14. 10.1016/j.molcel.2011.08.018 21925379 PMC3199020

[11] Zhan Y, Chen Z, Li Y, He A, He S, Gong Y, Li X, Zhou L. Long non-coding RNA DANCR promotes Malignant phenotypes of bladder cancer cells by modulating the miR-149/MSI2 axis as a ceRNA. J Exp Clin Cancer Res. 2018; 37:273. 10.1186/s13046-018-0921-1 30419948 PMC6233575

[12] Liu D, Li Y, Luo G, Xiao X, Tao D, Wu X, Wang M, Huang C, Wang L, Zeng F, Jiang G. LncRNA SPRY4-IT1 sponges miR-101-3p to promote proliferation and metastasis of bladder cancer cells through up-regulating EZH2. Cancer Lett. 2017; 388:281–91. 10.1016/j.canlet.2016.12.005 27998761

[13] Shih YP, Sun P, Wang A, Lo SH. Tensin1 positively regulates RhoA activity through its interaction with DLC1. Biochim Biophys Acta. 2015; 1853:3258–65. 10.1016/j.bbamcr.2015.09.028 26427649 PMC4621260

[14] Zhan Y, Liang X, Li L, Wang B, Ding F, Li Y, Wang X, Zhan Q, Liu Z. MicroRNA-548j functions as a metastasis promoter in human breast cancer by targeting Tensin1. Mol Oncol. 2016; 10:838–49. 10.1016/j.molonc.2016.02.002 26949125 PMC5423174

[15] Zhu Z, Wen Y, Xuan C, Chen Q, Xiang Q, Wang J, Liu Y, Luo L, Zhao S, Deng Y, Zhao Z. Identifying the key genes and microRNAs in prostate cancer bone metastasis by bioinformatics analysis. FEBS Open Bio. 2020; 10:674–88. 10.1002/2211-5463.12805 32027093 PMC7137804

[16] Zhou H, Zhang Y, Wu L, Xie W, Li L, Yuan Y, Chen Y, Lin Y, He X. Elevated transgelin/TNS1 expression is a potential biomarker in human colorectal cancer. Oncotarget. 2017; 9:1107–13. 10.18632/oncotarget.23275 29416680 PMC5787423

[17] Zhou HM, Fang YY, Weinberger PM, Ding LL, Cowell JK, Hudson FZ, Ren M, Lee JR, Chen QK, Su H, Dynan WS, Lin Y. Transgelin increases metastatic potential of colorectal cancer cells in vivo and alters expression of genes involved in cell motility. BMC Cancer. 2016; 16:55. 10.1186/s12885-016-2105-8 26847345 PMC4741053

[18] Martuszewska D, Ljungberg B, Johansson M, Landberg G, Oslakovic C, Dahlbäck B, Hafizi S. Tensin3 is a negative regulator of cell migration and all four tensin family members are downregulated in human kidney cancer. PLoS One. 2009; 4:e4350. 10.1371/journal.pone.0004350 19194507 PMC2632886

[19] Yam JW, Ko FC, Chan CY, Yau TO, Tung EK, Leung TH, Jin DY, Ng IO. Tensin2 variant 3 is associated with aggressive tumor behavior in human hepatocellular carcinoma. Hepatology. 2006; 44:881–90. 10.1002/hep.21339 17006924

[20] Chen H, Duncan IC, Bozorgchami H, Lo SH. Tensin1 and a previously undocumented family member, tensin2, positively regulate cell migration. Proc Natl Acad Sci USA. 2002; 99:733–38. 10.1073/pnas.022518699 11792844 PMC117374

[21] Wong CM, Tsang FH, Ng IO. Non-coding RNAs in hepatocellular carcinoma: molecular functions and pathological implications. Nat Rev Gastroenterol Hepatol. 2018; 15:137–51. 10.1038/nrgastro.2017.169 29317776

[22] Yuan JH, Yang F, Wang F, Ma JZ, Guo YJ, Tao QF, Liu F, Pan W, Wang TT, Zhou CC, Wang SB, Wang YZ, Yang Y, et al. A long noncoding RNA activated by TGF-β promotes the invasion-metastasis cascade in hepatocellular carcinoma. Cancer Cell. 2014; 25:666–81. 10.1016/j.ccr.2014.03.010 24768205

[23] Bussemakers MJ, van Bokhoven A, Verhaegh GW, Smit FP, Karthaus HF, Schalken JA, Debruyne FM, Ru N, Isaacs WB. DD3: a new prostate-specific gene, highly overexpressed in prostate cancer. Cancer Res. 1999; 59:5975–79. 10606244

[24] Haese A, de la Taille A, van Poppel H, Marberger M, Stenzl A, Mulders PF, Huland H, Abbou CC, Remzi M, Tinzl M, Feyerabend S, Stillebroer AB, van Gils MP, Schalken JA. Clinical utility of the PCA3 urine assay in European men scheduled for repeat biopsy. Eur Urol. 2008; 54:1081–88. 10.1016/j.eururo.2008.06.071 18602209

[25] Wei JT, Feng Z, Partin AW, Brown E, Thompson I, Sokoll L, Chan DW, Lotan Y, Kibel AS, Busby JE, Bidair M, Lin DW, Taneja SS, et al. Can urinary PCA3 supplement PSA in the early detection of prostate cancer? J Clin Oncol. 2014; 32:4066–72. 10.1200/JCO.2013.52.8505 25385735 PMC4265117

[26] Kim J, Piao HL, Kim BJ, Yao F, Han Z, Wang Y, Xiao Z, Siverly AN, Lawhon SE, Ton BN, Lee H, Zhou Z, Gan B, et al. Long noncoding RNA MALAT1 suppresses breast cancer metastasis. Nat Genet. 2018; 50:1705–15. 10.1038/s41588-018-0252-3 30349115 PMC6265076

[27] Mendell JT. Targeting a long noncoding RNA in breast cancer. N Engl J Med. 2016; 374:2287–89. 10.1056/NEJMcibr1603785 27276568

[28] Martens-Uzunova ES, Böttcher R, Croce CM, Jenster G, Visakorpi T, Calin GA. Long noncoding RNA in prostate, bladder, and kidney cancer. Eur Urol. 2014; 65:1140–51. 10.1016/j.eururo.2013.12.003 24373479

[29] Wang XS, Zhang Z, Wang HC, Cai JL, Xu QW, Li MQ, Chen YC, Qian XP, Lu TJ, Yu LZ, Zhang Y, Xin DQ, Na YQ, Chen WF. Rapid identification of UCA1 as a very sensitive and specific unique marker for human bladder carcinoma. Clin Cancer Res. 2006; 12:4851–58. 10.1158/1078-0432.CCR-06-0134 16914571

[30] Wang F, Li X, Xie X, Zhao L, Chen W. UCA1, a non-protein-coding RNA up-regulated in bladder carcinoma and embryo, influencing cell growth and promoting invasion. FEBS Lett. 2008; 582:1919–27. 10.1016/j.febslet.2008.05.012 18501714

[31] Yang C, Li X, Wang Y, Zhao L, Chen W. Long non-coding RNA UCA1 regulated cell cycle distribution via CREB through PI3-K dependent pathway in bladder carcinoma cells. Gene. 2012; 496:8–16. 10.1016/j.gene.2012.01.012 22285928

[32] Tan J, Qiu K, Li M, Liang Y. Double-negative feedback loop between long non-coding RNA TUG1 and miR-145 promotes epithelial to mesenchymal transition and radioresistance in human bladder cancer cells. FEBS Lett. 2015; 589:3175–81. 10.1016/j.febslet.2015.08.020 26318860

[33] Liu Q, Liu H, Cheng H, Li Y, Li X, Zhu C. Downregulation of long noncoding RNA TUG1 inhibits proliferation and induces apoptosis through the TUG1/miR-142/ZEB2 axis in bladder cancer cells. Onco Targets Ther. 2017; 10:2461–71. 10.2147/OTT.S124595 28503069 PMC5426477

[34] Du S, Hu W, Zhao Y, Zhou H, Wen W, Xu M, Zhao P, Liu K. Long non-coding RNA MAGI2-AS3 inhibits breast cancer cell migration and invasion via sponging microRNA-374a. Cancer Biomark. 2019; 24:269–77. 10.3233/CBM-182216 30883342 PMC13082510

[35] Yin Z, Ma T, Yan J, Shi N, Zhang C, Lu X, Hou B, Jian Z. LncRNA MAGI2-AS3 inhibits hepatocellular carcinoma cell proliferation and migration by targeting the miR-374b-5p/SMG1 signaling pathway. J Cell Physiol. 2019; 234:18825–36. 10.1002/jcp.28521 30924168

[36] Hall EH, Daugherty AE, Choi CK, Horwitz AF, Brautigan DL. Tensin1 requires protein phosphatase-1alpha in addition to RhoGAP DLC-1 to control cell polarization, migration, and invasion. J Biol Chem. 2009; 284:34713–22. 10.1074/jbc.M109.059592 19826001 PMC2787334

