# Supplementary material for: LncRNA MAGI2-AS3 inhibits bladder cancer progression by targeting the miR-31-5p/TNS1 axis
Source: Aging (Albany NY). 2020 Nov 20;12(24):25547–63. doi: 10.18632/aging.104162 (PMC7803496; doi:10.18632/aging.104162)
Supplement: Supplementary Tables [file aging-12-104162-s001.pdf]

SUPPLEMENTARY TABLES

Supplementary Table 1. Top 20 up-regulated and down-regulated lncRNAs.

Upregulated DElncRNA				Downregulated DElncRNA			
symbol	logFC	AveExpr	PValue	symbol	logFC	AveExpr	PValue
FIRRE	4.24307	0.04409	1.97E-13	FENDRR	-4.75748	1.72341	2.27E-46
IGFL2-AS1	3.95481	0.13467	1.35E-05	MIR100HG	-3.81192	1.87545	8.18E-36
U62317.1	3.33238	0.24628	1.65E-04	MBNL1-AS1	-3.03259	1.41488	8.11E-38
DUXAP8	3.32672	1.41590	4.42E-06	PART1	-3.01791	0.06381	2.32E-12
AC108860.2	2.70064	1.01530	1.42E-07	COLCA1	-2.68681	2.40232	2.85E-09
AP005230.1	2.57374	0.14437	3.01E-07	MAGI2-AS3	-2.46679	2.01071	2.84E-24
AP000251.1	2.56461	0.02993	1.51E-08	LINC00865	-2.39952	2.03987	5.56E-20
KCNMB2-AS1	2.53338	0.79135	1.95E-04	PWAR6	-2.38141	0.66409	3.79E-12
AP006284.1	2.51452	1.00227	5.41E-07	MEG3	-2.36711	3.16603	1.51E-14
AL049555.1	2.51237	3.75880	2.16E-05	AL357033.4	-2.07538	0.20694	3.69E-11
TRPM2-AS	2.41883	0.38014	1.79E-03	MIR22HG	-2.06559	4.05723	1.15E-22
AC011503.2	2.39476	0.31127	1.81E-08	AL645608.1	-2.04217	0.11579	1.93E-05
AC004816.1	2.38844	1.30984	2.45E-11	FZD10-AS1	-2.02980	0.81062	3.30E-07
AC099850.3	2.31535	0.86158	4.83E-09	DNM3OS	-2.01643	0.48396	1.37E-10
AC008105.1	2.30898	0.05912	7.27E-12	AP002884.3	-1.99364	0.42794	7.59E-11
AL390719.2	2.28764	1.79547	8.89E-04	EMX2OS	-1.92073	0.43802	1.94E-03
LINC01748	2.26231	0.48306	5.04E-05	SNHG14	-1.91885	3.75822	1.61E-09
FAM83A-AS1	2.23403	0.14461	2.45E-03	HOTTIP	-1.91724	0.32100	1.33E-04
AC245041.2	2.22507	2.45881	2.60E-03	AC093010.3	-1.89157	2.75284	5.09E-26
MIR200CHG	2.22403	2.10509	4.38E-04	NR2F1-AS1	-1.86470	1.27347	2.30E-11

logFC = a gene with log2 value of the differential expression multiple value; AveExpr = average expression

Supplementary Table 2. Top 20 up-regulated and down-regulated miRNAs.

Upregulated DEmiRNA				Downregulated DEmiRNA			
symbol	logFC	AveExpr	PValue	symbol	logFC	AveExpr	PValue
miR-210-3p	4.62511	8.81510	5.46E-19	miR-133b	-5.15661	0.95207	4.25E-25
miR-141-3p	3.67858	9.82684	1.46E-15	miR-490-3p	-5.14494	1.15875	1.46E-18
miR-96-5p	3.57823	4.47326	4.28E-18	miR-1-3p	-4.81818	4.47591	1.25E-21
miR-519a-5p	3.42622	1.01182	3.95E-05	miR-1247-3p	-4.71968	3.47773	4.14E-17
miR-200a-3p	3.12854	8.28879	6.73E-10	miR-133a-3p	-4.71287	3.95183	7.40E-23
miR-767-5p	3.07132	2.70253	5.61E-03	miR-139-3p	-3.95206	3.04878	3.17E-40
miR-516a-5p	3.06436	1.58977	5.00E-04	miR-143-3p	-3.91458	16.49923	2.95E-22
miR-934	3.06209	3.10810	1.02E-04	miR-204-5p	-3.71530	1.10992	3.62E-17
miR-429	3.00884	7.48660	3.04E-09	let-7c-5p	-3.56354	8.99740	3.39E-17
miR-33a-5p	2.95692	4.10462	2.52E-15	miR-30a-3p	-3.45495	10.87429	2.49E-31
miR-183-5p	2.84682	12.99602	4.88E-16	miR-145-3p	-3.37814	5.26756	3.97E-22
miR-18a-5p	2.75588	4.45054	4.61E-20	miR-99a-5p	-3.30306	7.01569	8.03E-13
miR-301a-5p	2.75011	2.05427	2.53E-14	miR-125b-2-3p	-3.30119	2.01884	4.10E-14
miR-205-5p	2.73992	12.16053	1.68E-06	miR-30c-2-3p	-3.01287	4.27366	3.26E-28
miR-301b-3p	2.55557	0.80324	9.05E-12	miR-1247-5p	-2.97500	2.30786	3.72E-09
miR-31-5p	2.55192	4.47895	6.55E-04	miR-145-5p	-2.92133	10.81009	1.05E-16
miR-135b-5p	2.48404	3.37284	6.76E-05	miR-139-5p	-2.88023	5.15249	1.67E-25
miR-200a-5p	2.48176	9.22886	4.90E-08	miR-100-5p	-2.85090	11.02392	5.35E-14
miR-1307-5p	2.47297	7.72021	3.37E-18	miR-195-5p	-2.78646	4.29438	9.77E-25
miR-33b-5p	2.45241	0.65998	7.29E-11	let-7c-3p	-2.61294	1.41547	7.53E-11

logFC = a gene with log2 value of the differential expression multiple value; AveExpr = average expression.

Supplementary Table 3. Top 20 up-regulated and down-regulated mRNAs.

Upregulated DEmRNA				Downregulated DEmRNA			
**Symbol**	**logFC**	**AveExpr**	**PValue**	**symbol**	**logFC**	**AveExpr**	**PValue**
CST1	5.40285	1.03505	8.65E-08	DES	-7.27215	4.54798	1.37E-12
CASP14	4.72295	1.77703	1.24E-03	ACTC1	-7.00399	0.64677	3.20E-36
ESM1	4.50803	2.71805	2.32E-12	C7	-6.58702	1.07344	3.23E-47
OTX1	4.38121	1.66218	9.79E-21	MYH11	-6.49746	6.26024	1.21E-14
COL10A1	4.32516	1.75621	8.08E-05	HSPB6	-6.26895	2.09592	2.23E-54
SLC1A6	4.31725	0.34745	4.41E-04	PGM5	-6.02556	2.17385	2.40E-47
KIF18B	4.14447	4.24221	3.00E-15	CNN1	-5.90860	4.29404	8.73E-22
MYBL2	4.04362	6.01672	1.56E-11	DPT	-5.68564	0.76314	9.27E-39
UBE2C	3.99162	5.92403	8.13E-16	ACTG2	-5.49885	5.59663	3.34E-14
TERT	3.96694	0.09974	4.51E-13	SYNM	-5.48402	3.87424	2.00E-34
TROAP	3.84596	4.44012	1.06E-14	MYOCD	-5.41293	0.46451	6.57E-57
CST6	3.82402	2.66890	1.27E-04	SYNPO2	-5.36335	4.33735	2.71E-29
MMP11	3.79990	5.49754	1.27E-04	FLNC	-5.33017	3.81797	8.40E-25
COL11A1	3.77211	2.48820	4.95E-04	NCAM1	-5.31444	0.89859	2.85E-50
NXPH4	3.76362	1.53380	2.13E-07	ADGRD1	-5.14387	0.23021	1.86E-49
KLC3	3.64595	2.19663	1.71E-07	LMOD1	-5.13507	3.77140	4.14E-31
NEK2	3.59185	3.92513	3.91E-14	CLEC3B	-5.09031	0.79048	1.05E-90
MNX1	3.58417	0.33982	1.03E-09	FAM107A	-5.07570	1.23285	2.38E-70
CENPA	3.58329	3.00805	3.80E-14	TNXB	-5.04425	2.08802	4.35E-58
IQGAP3	3.58136	4.99493	1.85E-13	P2RX1	-4.93366	0.53784	1.21E-44

logFC = a gene with log2 value of the differential expression multiple value; AveExpr = average expression.

Supplementary Table 4. Target miRNAs of MAGI2-AS3.

miRNA	geneName	clipExpNum
hsa-mir-31-5p	MAGI2-AS3	6
hsa-miR-519c-3p	MAGI2-AS3	5
hsa-miR-519b-3p	MAGI2-AS3	5
hsa-miR-519a-3p	MAGI2-AS3	5
hsa-miR-526b-5p	MAGI2-AS3	4
hsa-miR-3611	MAGI2-AS3	4
hsa-miR-142-3p	MAGI2-AS3	3
hsa-miR-374a-5p	MAGI2-AS3	3
hsa-miR-374b-5p	MAGI2-AS3	3
hsa-miR-513a-5p	MAGI2-AS3	2
hsa-miR-361-5p	MAGI2-AS3	2
hsa-miR-144-5p	MAGI2-AS3	2
hsa-miR-520a-5p	MAGI2-AS3	2
hsa-miR-525-5p	MAGI2-AS3	2
hsa-miR-3163	MAGI2-AS3	2
hsa-miR-506-5p	MAGI2-AS3	2
hsa-miR-382-3p	MAGI2-AS3	2
hsa-miR-224-5p	MAGI2-AS3	2
hsa-miR-452-5p	MAGI2-AS3	2
hsa-miR-4676-3p	MAGI2-AS3	2

clipExpNum = number of supported AGO CLIP-seq experiments (represent level of stringency).

Supplementary Table 5. miRNAs targeting TNS1.

miRNAname	geneName	clipExpNum
hsa-miR-5579-3p	TNS1	8
hsa-miR-21-5p	TNS1	7
hsa-miR-223-3p	TNS1	7
hsa-miR-590-5p	TNS1	7
hsa-miR-654-3p	TNS1	6
hsa-miR-374a-5p	TNS1	5
hsa-miR-410-3p	TNS1	5
hsa-miR-656-3p	TNS1	5
hsa-miR-374b-5p	TNS1	5
hsa-miR-20a-5p	TNS1	4
hsa-mir-31-5p	TNS1	4
hsa-miR-96-5p	TNS1	4
hsa-miR-103a-3p	TNS1	4
hsa-miR-107	TNS1	4
hsa-miR-148a-3p	TNS1	4
hsa-miR-181a-5p	TNS1	4
hsa-miR-181b-5p	TNS1	4
hsa-miR-181c-5p	TNS1	4
hsa-miR-182-5p	TNS1	4
hsa-miR-217	TNS1	4

clipExpNum = number of supported AGO CLIP-seq experiments (represent level of stringency).
